# Self-management for obesity and cardio-metabolic fitness: Description and evaluation of the lifestyle modification program of a randomised controlled trial

**DOI:** 10.1186/1479-5868-5-53

**Published:** 2008-10-27

**Authors:** Tahna L Pettman, Gary MH Misan, Katherine Owen, Kate Warren, Alison M Coates, Jonathan D Buckley, Peter RC Howe

**Affiliations:** 1 Australian Technology Network (ATN) Centre for Metabolic Fitness and Nutritional Physiology Research Centre, School of Health Sciences, University of South Australia, Adelaide 5000, South Australia, Australia; 2 Spencer Gulf Rural Health School, c/o University of South Australia, Nicolson Avenue Whyalla 5608, South Australia, Australia; 3 Centre for Health Economics Research and Evaluation (CHERE), University of Technology Sydney, Broadway 2007, UK

## Abstract

**Background:**

Sustainable lifestyle modification strategies are needed to address obesity and cardiovascular risk factors. Intensive, individualised programs have been successful, but are limited by time and resources. We have formulated a group-based lifestyle education program based upon national diet and physical activity (PA) recommendations to manage obesity and cardio-metabolic risk factors. This article describes the content and delivery of this program, with information on compliance and acceptability.

**Methods:**

Overweight/obese adults (*n *= 153) with metabolic syndrome were recruited from the community and randomly allocated to intervention (INT) or control (CON). Written copies of Australian national dietary and PA guidelines were provided to all participants. INT took part in a 16-week lifestyle program which provided a curriculum and practical strategies on 1) dietary and PA information based on national guidelines, 2) behavioural self-management tools, 3) food-label reading, supermarkets tour and cooking, 4) exercise sessions, and 5) peer-group support. Compliance was assessed using attendance records and weekly food/PA logs. Participants' motivations, perceived benefits and goals were assessed through facilitated discussion. Program acceptability feedback was collected through structured focus groups.

**Results:**

Although completion of weekly food/PA records was poor, attendance at information/education sessions (77% overall) and exercise participation (66% overall) was high, and compared with CON, multiple markers of body composition and cardio-metabolic health improved in INT. Participants reported that the most useful program components included food-label reading, cooking sessions, and learning new and different physical exercises, including home-based options. Participants also reported finding self-management techniques helpful, namely problem solving and short-term goal setting. The use of a group setting and supportive 'peer' leaders were found to be supportive. More frequent clinical assessment was suggested for future programs.

**Conclusion:**

This group-based lifestyle program achieved improvements in body composition and cardio-metabolic and physical fitness similar to individualised interventions which are more resource intensive to deliver. It confirmed that active training in lifestyle modification is more effective than passive provision of guidelines. Such programs should include social support and self-management techniques. Continued clinical follow up may be required for long-term maintenance in individuals attempting lifestyle behaviour change. Program facilitation by peers may help and should be further investigated in a community-based model.

## Background

A randomised, controlled, parallel group intervention trial aimed at improving body composition and cardio-metabolic risk factors was conducted in free-living adults with metabolic syndrome (MetS). The study used a group-based lifestyle modification program of non-energy restricted diet and physical activity (PA) intervention, based on the Australian national guidelines for diet and physical activity (PA). The intervention involved 103 participants and resulted in improvements in body composition, blood pressure, blood lipid levels, physical fitness and dietary intake, the details of which have been described elsewhere [1]. The purpose of this article is to provide a detailed description of the intervention used in the study, and to report on the acceptability of the program and the practical implications of applying national recommendations for lifestyle change.

### Rationale

Obesity continues to be a major public health issue, and central obesity and MetS are also becoming increasingly prevalent. In Australia, approximately 60% of the population are overweight or obese [2], and 30% have central obesity. Increasing levels of obesity, in particular central obesity, are also driving an increase in the prevalence of MetS [3]. Consensus on the long-term effectiveness of intervention programs for reducing obesity has not been established [4]. Similarly, there is no one agreed treatment for MetS [5]. Despite this, lifestyle intervention (weight loss, dietary improvement and PA) is emphasised as first-line therapy for these conditions. National guidelines for appropriate diet and PA are available, but the uptake of these recommendations is unknown.

Current literature suggests that significant effort is required to achieve sustained changes in body composition and metabolic health. Lifestyle intervention combining intensive dietary modification with prescribed levels of PA can reduce the incidence of chronic disease [6,7] but a range of factors can negatively influence the successful adoption and maintenance of positive lifestyle changes [8], resulting in difficulty improving lifestyle behaviours. Consequently, many programs demonstrate short term weight loss, for example at 3–6 months, but few show success in maintaining weight loss at 12 months or beyond [9-11] Sustained weight loss and modification of risk factors has been achieved following long-term intensive interventions [7,12] but these methods generally rely on prescribed diet and exercise together with counselling and supervision by dietitians, counsellors, physical trainers, psychologists and behaviourists. The use of highly prescriptive diet and exercise regimes may present difficulties for some people to integrate into their existing lifestyle, and the use of health professionals to work one-on-one with individuals in order to effectively implement this type of program can make such approaches cost and time intensive, and potentially unsustainable at the community level. Evaluations of primary-care based programs and applications of national lifestyle guidelines appear to be practical [13-17], and recent evidence suggests that they may be effective [18-20] but again these types of programs rely heavily on individualised intervention, making their delivery expensive.

The approach we are evaluating in this study involves a program conducted in a group setting, incorporating some of the potentially beneficial aspects of self-management programs (SMP). SMP appear to be effective for chronic disease management [21-23] and might also be useful for the prevention of chronic disease [24-26]. Key features of such programs include shared goal-setting, lifestyle skills teaching, ongoing follow-up and support [27] as well as promoting the establishment of peer support networks, which have been suggested as a useful tool for improving compliance with interventions for weight loss [28] and diabetes prevention [29]. Delivery of this program in a group setting rather than on an individual basis was aimed at reducing the resources required for the delivery of the program.

## Methods

### Clinical study design

Volunteers were recruited from Whyalla, a regional community of South Australia with rates of overweight and obesity which exceed the State average. Adults in the community responded to media advertisements calling for volunteers to participate in the *Shape up for Life *lifestyle intervention trial which was aimed at changing lifestyle habit to reduce obesity and improve health. Beginning in 2006, 153 volunteers meeting international criteria [30] for MetS were enrolled (111 females, 42 males; aged 45.1 ± 0.8 years). Participants weighed on average at baseline 102.6 ± 2.3 kg, and mean BMI was 36.6 ± 0.7. Mean waist circumference was 115 ± 1 cm. Individuals taking medication for blood pressure, blood lipids or with a diagnosis of diabetes mellitus (type 1 or type 2) were excluded. Eligible participants were randomly allocated to intervention (INT; *n *= 103); or control (CON; *n *= 50). There were no significant differences in baseline characteristics between INT and CON. Assessments of anthropometry, body composition, cardiovascular and metabolic health, physical fitness, dietary intake, PA and quality of life were performed at baseline and after 4 and 12 months. The clinical study protocol was approved by the Human Research Ethics Committee of the University of South Australia, and all study participants provided written informed consent prior to participation.

### Lifestyle intervention

All participants were provided with standard booklets of the Australian guidelines for healthy eating [31] and PA [32]. No other intervention was provided to CON. A structured 16 week information and education program and group exercise was conducted for INT. The program, called '*Shape up for Life'*, ran as group sessions for 2 hours each week.

### Key features of the program

The program sessions were framed to translate the various recommendations and advice contained in National guideline documents into 'real life'. Practical strategies were used to aid in the uptake of dietary and physical activity recommendations, given that people are concurrently managing family, work and study; may have limited finances; may be travelling away from home frequently, and may work long and irregular hours (eg. split shifts and night shifts). Sessions were held at different times of the day/night and on different days/nights of the week, to accommodate participants' family and work commitments.

#### Dietary and physical activity information

Dietary topics covered during information/education sessions are summarised in Table 1. Information for these sessions was drawn from several resources including the Australian Guide to Healthy Eating [33] (AGHE), Nutrition Australia 'Healthy living book'[34] and factsheets [35] and Glycemic index (GI) Australian resources [33]. Dietary advice reflected what is advocated by the Australian dietary guidelines (ADG)[36], including advice to balance energy intake (*ad libitum*) with energy expenditure through daily activity. Participants were not given meal plans or individualised diet counselling, and energy targets were not prescribed. Specific macronutrient and micronutrient requirements were not discussed, except for general recommendations to reduce saturated fat, sodium and alcohol (consistent with ADG). Rather, quality and variety of dietary intake were emphasised. Emphasis was placed upon improving dietary quality through the intake of more nutrient dense foods (that are inherently less energy dense), replacing refined carbohydrates with low GI and fibre-rich options, replacing sources of saturated fats with polyunsaturated fats, reducing salt/sodium intake, and increasing food variety from recommended food groups (mainly vegetables, fruits, grains, nuts and seeds). Participants were encouraged to moderate their intake of alcohol and caffeine, and to drink more water. Health benefits of the standard dietary recommendations were also discussed, including the use of lower GI foods for regulation of blood glucose and satiety; and omega-3 fats for cardiovascular benefit and body fat reduction [37]. Some of the practical issues regarding food choices were also discussed, including shopping on a budget, incorporating meat alternatives (if desired), modifying recipes to make healthier meals, trying new foods on family/children, and choosing more appropriate 'fast food' options.

**Table 1:** Activities in the *Shape up for Life *program

**Dietary topic**	**Activity description**
***Healthy eating & Energy Balance***	Introduction to healthy eating – variety, quality; Balancing energy intake with expenditure; Energy and Nutrient density of food; Barriers to eating healthily (food triggers), problem solve common issues
***Glycemic index (GI)***	Background; Using GI for choosing better quality CHO; Reducing refined CHO
***Food labels***	Judging food quality; Identifying low fat/low sugar options based upon 1) nutrition info panel 2) ingredients list 3) nutrition claims
***Fats***	Recommended saturated fat intake; Types of fat; Choosing better quality fats, Benefits of omega-3 polyunsaturated fatty acids and plant sterols
***Carbohydrate & Fibre***	Benefits of fibre; Disadvantages of low CHO/low fibre intake; Increasing fibre
***Salt***	Disadvantages of salt; Recommended sodium intake; Food sources; Brainstorm alternatives for flavouring food without salt
***Shopping tips***	Healthy eating on a budget
***Refresher: GI, Fat & Salt***	Recap and feedback on GI/CHO-Fat-Salt; What barriers participants have found, problem-solve common issues
***Food variety***	Importance of variety; Food variety checklist (Nutrition Australia fact sheet)
***Takeaway food***	Disadvantages of excess takeaway; Brainstorm alternatives
***Alcohol & Caffeine***	Disadvantages of excess alcohol & caffeine; Brainstorm alternatives
***Supermarket 'secrets'***	Judging quality, price and processing of food

**Physical activity topic**	**Activity description**

***Exercise & Energy Balance***	Introduction to exercise – Endurance, Resistance, Flexibility; Using pedometer and physical activity diary
***Endurance exercise1***	Moderate intensity exercise; FIT formula (Frequency, Intensity, Time); monitoring exercise intensity (Talk-test, RPE, HR)
***Resistance training1***	Benefits of strength training; Common questions; Using resistance bands
***Flexibility exercise***	Importance of stretching; How to stretch safely and effectively
***Endurance exercise2***	Recap and Feedback on exercise; Increasing variety by adding to FITT formula (Frequency, Intensity, Time, and *Type*)
***Resistance training2***	Benefits of building lean tissue; How to lift safely and effectively

PA topics are summarised in Table 1. Information regarding physical activity and exercise was drawn from several resources, including National PA guidelines, Active Australia 'getting started' booklet [38], Council on the Ageing (COTA) factsheets (accessed online ), and State government of Victoria consumer health fact sheets (accessed online ). Participants were not given specific targets other than to aim to achieve the PA recommendations of the national guidelines. Education topics included: why exercise is important; differentiation between the benefits of different types of exercise (cardiovascular, resistance, flexibility); benefits of moderate intensity aerobic activity; monitoring exercise intensity; using the FITT principle (Frequency, Intensity, Time, Type) to vary and increase activity; and the importance of incidental and transport-related activity. Options for incorporating resistance training into an exercise regimen were also discussed (and demonstrated), including the use of resistance bands, own body weight, dumbbells, bar-bells and resistance machines, highlighting the benefit of improving body composition through increasing lean tissue (fat-free) mass. Participants in INT were provided with free access to a gymnasium during the intervention period to assist with the adoption of exercise guidelines.

#### Behavioural lifestyle self-management

Lifestyle self-management tools used in the intervention are summarised in Table 2. We encouraged the use of strategies to address some of the barriers to adopting healthy lifestyle habits, achieved mainly by incorporating tools adapted from the Stanford chronic disease SMP, originally developed by Lorig and colleagues [39]. One of the essential features that we incorporated was short-term goal setting, or 'action planning', as described by the Stanford model, which teaches participants to break long term goals into smaller, more achievable (action-specific) steps. 'Triggers' affecting food and PA choices were discussed, using an activity adapted from the DPP *Lifestyle balance *core program, entitled "take charge of what's around you" [40]. Another activity called 'obesity prevention', which was inspired by concepts introduced by Swinburn and Egger [41], was used to raise awareness of external/environmental obesity-promoting factors (eg. technology, convenience food, limited fresh food access).

**Table 2:** Lifestyle self-management tools used in *Shape up for Life*

**Activity title**	**Delivery method**	**Description**	**Anticipated outcomes**
***Action Planning, Problem solving***	Leader facilitates sharing of information	Behaviour-specific short goal setting, addressing lifestyle barriers	Learn to set reasonable, behaviour-specific goals (shared or individual), and monitor personal goals/progress
***Managing food and activity triggers***	Group brainstorm, problem solving	Identify habits relating to diet and physical activity, selecting common issues to address.	Learn coping strategies for personal dietary issues (eg. snacking), and for increasing opportunities for physical activity
***Managing family, social and cultural pressures***	Group brainstorm, problem solving	Identify habits associated with social situations and cultural/family habits, selecting common issues to address	Learn coping strategies for habitual overeating and drinking, and for increasing opportunities for physical activity
***Progressive muscle relaxation***	Leader reads and directs scripted activity	Guided practice of relaxation of major muscle groups	Raise body/muscle awareness to assist in movement patterns (eg. for exercise), and general tension release
***Better breathing***	Leader provides lecturette & reads script for activity	Guided practice of breathing fully and more effectively	Learn to breathe diaphragmatically, to assist in physical activity, and for relieving shortness of breath, and general tension
***Fatigue management***	Leader provides lecturette, group brainstorm	Identify causes of, and solutions for, managing fatigue	Learn coping strategies to address low energy, to improve motivation; in general and for physical activity
***Self talk (positive thinking)***	Leader provides lecturette, group brainstorm	Identify personal negative thoughts, and how to change into positive, constructive thought patterns	Learn to use helpful thoughts for motivation, improve self-efficacy to reach goals
***Depression management***	Leader provides lecturette, group brainstorm	Identify causes of, and suggestions for managing difficult times in life	Learn coping strategies to assist in managing for motivation, improve self-efficacy to achieve and maintain lifestyle changes
***Mind management and distraction***	Leader provides lecturette & reads script for activity	Guided practice of cognitive short-term distraction techniques	Learn to use distracting thoughts to assist in managing behavioural issues, self-control, (eg. for eating and physical activity) and stress
***Making informed treatment decisions***	Leader provides lecturette, group brainstorm	Discussion about health treatments (eg. weight loss products and programs) highlighting possible benefits, risks and efficacy	Raise overall awareness of issues relating to health-related treatments, highlighting need to make informed decisions about lifestyle choices
***Guided imagery***	Leader reads script for activity	Guided practice of cognitive relaxation sequence	Learn to use relaxing thoughts to assist in relaxation, relieve stress and tension
***Obesity prevention***	Leader provides lecturette, group brainstorm	Discussion of hosts, carriers and environments contributing to obesity, suggestions for change	Raise overall awareness of the modifiable social, environmental and individual influences on lifestyle

**Figure 1:**
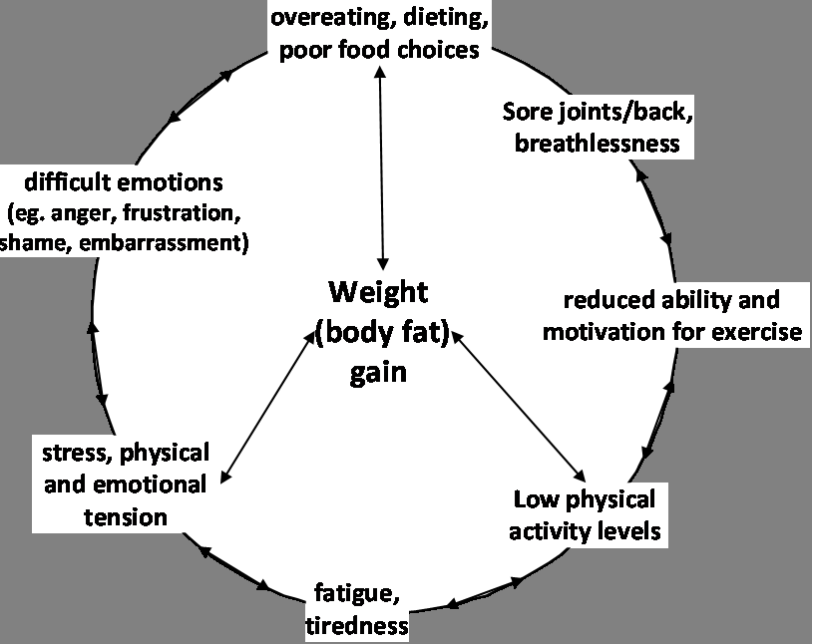
Diagrammatic representation of the 'vicious cycle' used throughout the Shape up for Life program.

Another concept that was introduced and referred to throughout the program was a diagrammatic concept of the 'vicious cycle' (Figure 1) in overweight/obesity, which was based upon the 'symptom cycle' described in the Stanford chronic disease SMP. This was intended to illustrate both the negative *and *positive impact that people can have on their own health behaviour and weight (body fat) management. In the chronic disease context, the cycle is used to highlight that symptoms experienced are not due to only one cause (the disease) but interactions of various other symptoms (for example; stress can cause tense muscles which may cause pain). For our purpose, factors that can contribute to weight (body fat) gain were included, and self-management strategies to address these issues were highlighted. For example; it was suggested that fatigue/tiredness can reduce motivation for exercise, which can lead to loss of fitness and de-conditioning, potentially increasing risk of pain and injury when exercising, and negatively self-perpetuating the 'cycle'. It was also posed that secondary behaviours may arise from an existing issue or problem; for example fatigue/tiredness/stress may lead to non-hungry eating or poor food choices (e.g. energy-dense snacks or fast food). The alternative focus of the cycle we proposed was that *healthy *behaviours can have a *positive *impact on other lifestyle factors, for example; managing sore joints through appropriate exercise can increase flexibility and improve ability, which can boost motivation for PA and in turn impact positively upon other issues through positive feedback. Further description on the adaptation of self-management activities from the Stanford course may be found elsewhere [42].

#### Food labels, shopping and cooking

To demonstrate 'real-life' dietary and activity changes, practical activities were conducted. These practical activities included food-label reading education using real product labels, and referring participants to Nutrition Australia recommendations for understanding food labels [34,43]. Participants were encouraged to bring in labels from their own shopping to discuss. A visit to a local supermarket provided participants with another opportunity to review nutrient information labels, nutrient claims, and to physically compare different food products to determine which were better quality. In these interactive tours, participants were directed towards buying seasonal produce and alternatives such as frozen vegetables, dried beans, and tinned fruit. Participants were directed to judge for themselves the nutritional value of commonly-used products such as bakery and delicatessen items, savoury crackers, margarines and spreads, yoghurts and dairy desserts. Two interactive cooking sessions were conducted to demonstrate meal ideas consistent with education topics, and to encourage variation in the intake of foods, condiments and cooking methods. During these sessions participants were provided with recipes and ingredients to prepare various meals, snacks and desserts. A cookbook was later compiled and provided to participants, including the recipes individuals contributed during sessions.

#### Exercise sessions

Participants were asked to attend at least one supervised exercise session each week during the intervention period. Group exercise was conducted for 45–60 minutes, usually directly after the information session. Additional sessions were held at different times to accommodate participants' work/family commitments. Study leaders demonstrated techniques such as heart-rate monitoring for aerobic activity, safe and effective use of gym equipment, core strengthening, and stretches. Examples of other types of exercises included in the sessions were walk-aerobics [44], weight-room circuits, aerobics, step/dance-aerobics, hand-weights and resistance bands, mixed circuits, and Tai Chi. Gymnasium facilities were established at the commencement of the study, shared with University staff and students, equipped with resistance and cardio machines, dumbbells, fitballs, boxing bags, mats and space for aerobics (with a TV and CD/DVD players). These facilities were made freely available to participants after they had been instructed in their correct use by study staff. Rubber resistance bands and handouts on exercise examples were offered for home use. Pedometers (step-counters) were also provided to encourage participants to monitor their movement through daily incidental activity. Participants were informed that a recommended level to work towards achieving was 10,000 steps per day, which appears to be in line with national recommendations [45-47]. Heart rate monitors were available during exercise sessions to encourage participants to monitor their cardiovascular exercise intensity.

#### Peer-group setting

Information sessions were presented by two leaders, and groups consisted of up to fifteen individuals per session. Several repeat sessions were held throughout the week to accommodate all participants. The intervention was conducted by a study coordinator with health science/nutrition background together with a leader with expertise and experience in chronic disease SMP facilitation and training. Both leaders assumed a 'peer' role in conducting sessions, in that they provided information for participants to consider incorporating into their existing lifestyles. Leaders encouraged all participants to modify their own dietary and PA behaviours. Participants were also encouraged to share their experiences and ideas, to listen to and be respectful of others in the group, and to interact with each other both during and outside of sessions.

### Additional program materials

The lifestyle program was compiled as a "leader's manual" which included written narratives, brainstorming activities, and prompts for leaders to 'model' lifestyle behaviours. PowerPoint™ slides were designed and used to guide session conduct. The manual ensured session consistency for the different intakes of study participants, and also served as a 'script', so that with training, individuals other than study coordinators would be able to lead future education sessions. Other supporting materials for the program included action-plan templates, a weekly food and PA record, information hand-outs, study newsletters, recipes and fact-sheets.

### Compliance assessments

Weekly attendance was recorded for each individual, for attendance at both information and PA sessions. If absent from information sessions, participants were followed up by phone to ascertain a reason and to schedule a 'catch up' prior to the next week's session, and were encouraged to do their own exercise if they were unable to attend group exercise (or did not wish to take part in group exercise). Sample foods were provided by sponsor companies to offer to participants as examples of healthy products (wholegrain breads, breakfast cereal, tinned fish and unsalted nuts). A template log was provided to participants to complete during the week to record their PA together with the number of serves of food products consumed.

### Participant feedback

Individuals' motivations for wanting to achieve lifestyle changes, and their perceptions of how much their lifestyle had changed were discussed during the information session at week 12. Questions were intended to prompt reflection on healthy habits adopted as a result of the program (being 3 months into the intervention), and also to focus on maintaining these changes (having only 1 month until the end of the intervention when physical assessments would be performed). Participants were asked why they were taking part in the study; and what changes they had made as a result. Participants' personal goals were collected at the end of the intervention at session week 16. Long-term goal-setting was conducted to encourage behaviour maintenance over the remaining 8 months of the study. Participant responses were transcribed verbatim and later categorised into common themes. Finally, feedback was invited from study participants after the 16-week program. Focus groups were conducted by researchers who were independent from the delivery of the study, with the aim of evaluating participants' satisfaction with the program. Examples of information gathered included (1) participants' opinions on the diet and PA components of the program, (2) how the program impacted on the participants/their families, (3) whether participation enabled sustainable lifestyle changes, and (4) suggestions for improvement of the program.

### Data analysis

Data were collated and analysed using MS Excel (Microsoft corporation, US) and SPSS 15.0 for Windows (SPSS Inc, Chicago, Illinois). Session attendance counts and weekly report completion scores were entered into spreadsheets and chi-square analyses were performed. Correlations (Spearman) were performed using SPSS. Data from weekly records (i.e. number of food serves; duration of PA) were not directly used, due to overall poor completion. However, the records received were assessed for completeness, and these scores were incorporated into the overall compliance assessment. Qualitative data (participant quotes) were transcribed verbatim and entered into spreadsheets, and subjective responses were reduced and grouped thematically to reveal predominant themes. Focus group data were recorded on audiotape, transcribed, and compiled thematically using evaluative codes.

## Results

### Clinical outcomes

**Figure 2:**
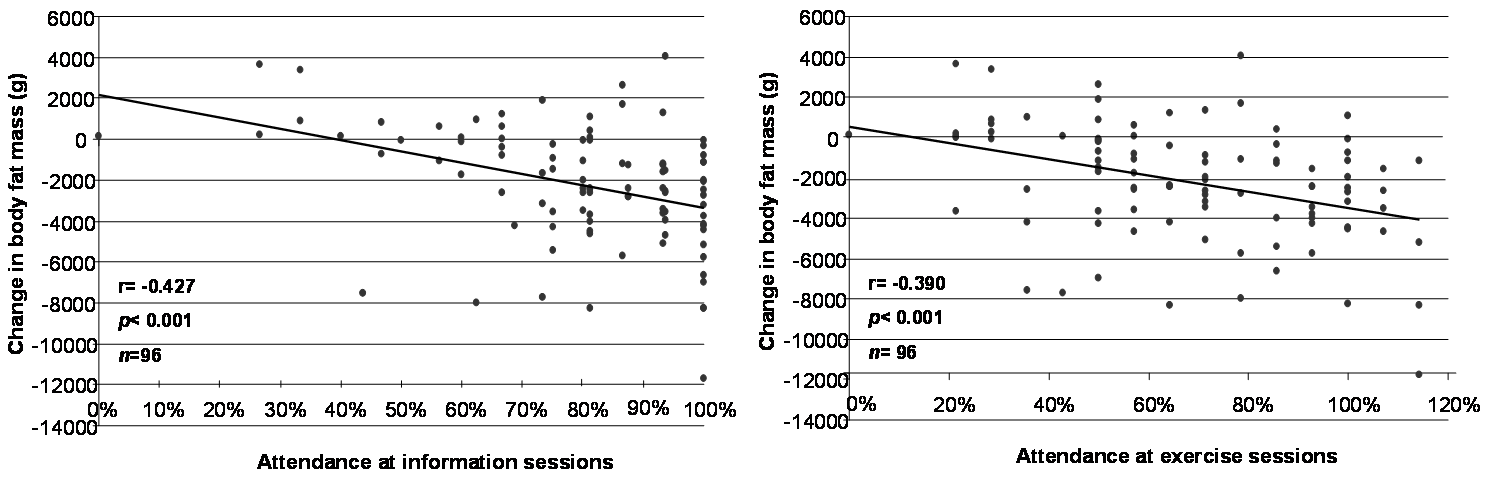
**Correlations between rate of attendance at intervention program sessions and reduction in body fat mass**. Note: Attendance at exercise exceeded 100% in some cases as participants chose to attend more sessions than required.

The success of the clinical study has been described elsewhere [1]. The study demonstrated that the intervention was effective in improving body composition (4% reduction in body fat mass and abdominal body fat mass, 3 cm waist reduction), blood pressure (>5% reduction), total cholesterol and LDL (8 – 10% reduction), and physical fitness (12% improvement). There were also statistically significant correlations within-individuals between attendance at program sessions and clinical changes from baseline. For example, greater attendance at information and exercise sessions was associated with greater reductions in body fat (Figure 2), blood pressure (DBP *r *= -0.31, *p *< 0.001; and SBP *r *= -0.28, *p *= 0.01), total cholesterol (*r *= -0.35, *p *< 0.001) and plasma glucose (*r *= -0.35, *p *< 0.001). Dietary intake and PA outcomes were limited by the methods of data collection; however both groups (intervention and control) significantly reduced overall energy intake and increased levels of PA (data not shown).

### Program compliance

**Figure 3:**
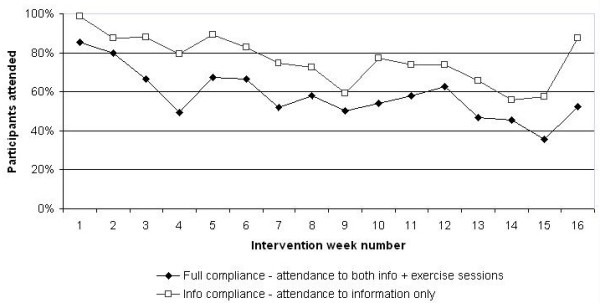
Attendance at intervention sessions (all participants, *n *= 103).

Information session attendance was 77%, with an average of 12 sessions attended out of a total of 15 or 16 scheduled (one program intake was condensed into 15 sessions due to the course overlapping Christmas). Exercise sessions were less attended at 66% overall, with an average of 9 sessions attended out of a possible 14 conducted (two sessions were not followed by exercise due to time constraints (cooking)). In most cases, the reasons for non-attendance to information/exercise sessions were related to work or family commitments (data not shown). Attendance declined gradually approximately half-way through the program (Figure 3). Based upon data from other interventions [15,48-50] and testing with chi-square; attendance to information sessions did not differ from an expected 75% (*p *= NS) but exercise sessions were in fact attended more than an expected 50% (*p *< 0.001).

Weekly participant diet and physical activity records were poorly maintained, with approximately one-third of the group not completing or not returning weekly records, and another third did not complete fully or accurately. Ultimately a subjective assessment was made by two research staff (one of whom was actively engaged in the program with the participants, and another who had no direct contact with participants) to judge the accuracy of returned records. A score was allocated by mutual agreement between the two research staff, of 0 = effectively non-compliant; 0.5 = uncertain or partly-compliant (eg. not attending exercise sessions, but reported doing additional PA outside of sessions); or 1.0 = appears compliant. The average of all scores was 0.51 (part-compliant). Attendance at exercise sessions were significantly correlated with attendance at information sessions. Weekly report completion scores also correlated significantly with information session attendance (Table 3).

**Table 3:** Correlation coefficients for attendance at sessions and compliance outcomes (Spearman) all participants (*n *= 103)

	**Exercise attendance**	**Information attendance**	**Weekly log completion score**	**Overall compliance score**	**Non-compliance score**
**Exercise attendance**	(1.00)	0.80**	0.63**	0.86**	-0.82**
**Information attendance**	0.80**	(1.00)	0.68**	0.87**	-0.99**
**Weekly reports completion score**	0.63**	0.68**	(1.00)	0.91**	-0.68**
**Overall compliance score**	0.86**	0.87**	0.91**	(1.00)	-0.87**
**Non compliance score**	-0.82**	-0.99**	-0.68**	-0.87**	(1.00)

** Correlation is significant at the 0.01 level (2-tailed).

### Participant perceptions

**Figure 4:**
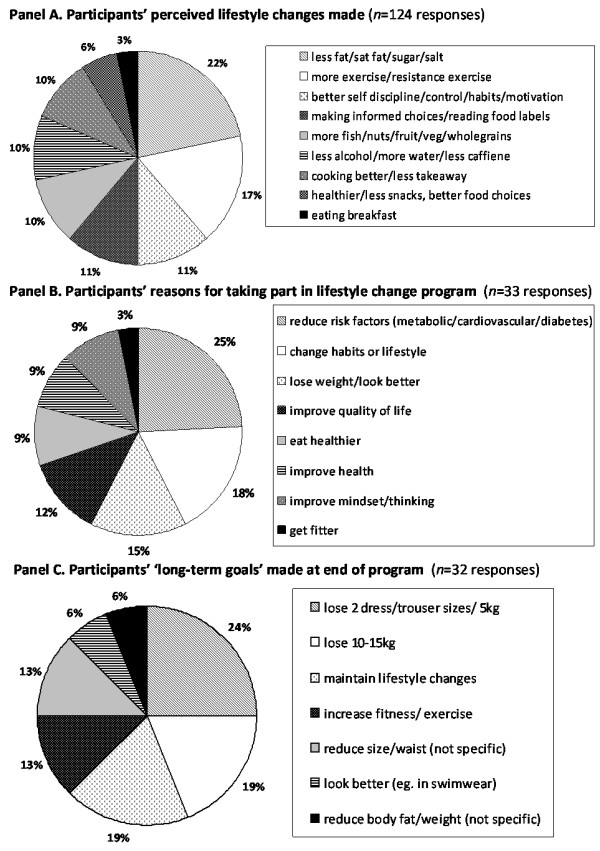
Participant responses during facilitated discussion.

A total of *n *= 124 responses were obtained from the group sessions (week 12 and week 16) where questions were posed (Figure 4). Reasons for taking part in the program (*n *= 33) were predominantly to reduce risk for diabetes, metabolic or cardiovascular disease (25%); to change lifestyle or habits (18%); and to lose weight or look better (15%). Other categories that emerged included improve quality of life; improve mindset/thinking; improve general health; eat healthier; and get fitter. Discussion of lifestyle habits changed elicited themes such as eating breakfast; healthier/less snacks or better food choices overall; drinking more water and/or less alcohol and caffeine; improved cooking and less takeaway purchase; inclusion of more fish, nuts, fruit, veg and wholegrains; more self-discipline, self control, motivation and better habits; making informed choices and reading food labels; more exercise and/or resistance exercise; and reduced fat/saturated fat, sugar and salt. The categories with the greatest responses were reduced fat/saturated fat, sugar and salt (22%) and more exercise and/or resistance exercise (17%). Personal goals made (*n *= 32) when prompted to consider making a goal at the end of the program included firstly to lose 2 dress/trouser sizes or 5 kg (24%); to maintain lifestyle changes (18%) and to lose 10–15 kg (18%).

### Program acceptability

Finally, feedback from study participants during focus groups was used evaluate overall satisfaction with the program. A total of 33 participants volunteered to take part in these focus groups. Questions and guided discussion revealed information on 3 common areas; food/dietary factors, exercise issues, and support. Participants reported that food-label reading and information on takeaway food were useful, together with cooking sessions. Participants were pleased that the program leaders took a peer role in all sessions, and had realistic expectations of them, particularly in exercise sessions. Home-based exercise options were viewed favourably; however participants reported needing follow-up instructions, such as use of resistance bands. Provision of the gym was appreciated (due to cost saved), but for many it was not utilised with the main reason reported for this lack of use being embarrassment.

Regarding overall program structure, participants felt that the leaders' encouraging attitudes assisted with compliance, and found goal-setting and problem solving skills useful. Home-based exercise options, monthly study newsletters, and importantly the commonalities amongst the peer group were also appreciated and considered to be useful. The group setting was found to be of a supportive nature, and new social supports were formed as a result.

A common suggestion made was to schedule more frequent clinical assessments, to maintain motivation and be aware of personal progress. It was suggested that this take place half-way through the intervention (week 8). Participants reported having experienced a feeling of loss at the end of the intervention. Some participants suggested that future programs include a referral pathway (e.g. to counsellor or psychologist) to deal with underlying issues causing barriers to behaviour change.

## Discussion

The intervention program evaluated in the current analysis was effective in improving a range of health outcomes in INT compared to CON and that are described elsewhere [1]. Despite an average of approximately two thirds of sessions attended overall, this was sufficient to improve diet, increase PA and produce clinically relevant outcomes in free-living individuals with MetS. This suggests that the methods and delivery of this program were acceptable. Also, the content and structure of the program has to some extent been reported as acceptable by participants.

### Program content

The content of the education program was based upon Australian national guidelines. There is recent evidence to suggest that lifestyle intervention in accordance with general lifestyle recommendations can be beneficial to health in at-risk individuals [15,18,20] when delivered by health professionals/lifestyle counsellors. In order to achieve clinically significant outcomes through our group-based approach, we hypothesised that promoting dietary quality would result in a more balanced, nutrient-dense diet, with increased complex carbohydrate, bioactive nutrient and fibre intake, together with reduced total energy intake. Additionally we hypothesised that benefit may be gained by introducing participants to the 'functional' aspects of recommended foods (low GI, omega-3), for which the evidence-base relating to health benefits is increasing [51-54]. Our intervention program provided a simple, group-based approach to improving diet and PA, and essentially provided a strategy for encouraging adoption of the national recommendations. Consequently this intervention may be more easily transferred into communities than complicated or restrictive diets and personalised or prescribed exercise, which are generally unsustainable.

The overall aim of the PA component of the intervention was to increase energy expenditure and promote overall fitness by increasing moderate/vigorous exercise and incidental/low intensity (long-duration) leisure activity in accordance with national guidelines. We encouraged engagement in regular PA in order to achieve not only body fat reduction, but also to improve risk factors which constitute the metabolic syndrome. [55,56]

We also proposed that self-management techniques, that have proven successful in the management of chronic disease, might also be applicable in managing overweight [26,57]. Lifestyle topics and strategies were incorporated to address some of the external and personal issues that are often barriers to healthy lifestyle choices. The activities incorporated were adapted from other successful SMP's described previously. The effectiveness of the Stanford techniques for chronic disease have been demonstrated internationally [21,58] and locally [59].

The literature suggests that programs combining diet, exercise, and behaviour modification are the most effective for improving health outcomes in the short term [60] (as cited in [61]). Self-managed strategies are also useful in the management of weight [26] and are considered to apply especially well to preventive interventions involving lifestyle modification [57]. In our program, practical sessions and supervised exercise were included to address 'real-life' aspects of dietary choice and PA, and to introduce participants to different options to consider. Self-efficacy is also recognised as associated with health behaviour change [62] and current evidence continues to confirm it's importance to diet and activity [63]. Therefore action planning and problem solving were integral to the program to promote self-efficacy; through accomplishing small, weekly behavioural goals which would lead to achieving a larger health-related goal.

### Program format and conduct

Recent data has indicated that group-based lifestyle intervention with monthly follow-up can improve body weight, PA, health and wellbeing to a similar extent as individualised dietetic treatment. [64] In our program, group meetings were conducted for all sessions. A group setting was used to more effectively manage a number of individuals over time, compared one-on-one individual interventions [64]. A similar strategy has also been recently described for engaging larger groups of individuals; through adaptation of the DPP intervention for delivery in the community [17]. Additionally there is evidence to show that the social support network may assist individuals in maintaining weight loss through continued follow-up [28], shared short-term goal setting and problem solving [26], which is also why we considered the peer-group setting to be a useful strategy. The presence of a peer leader endeavouring to achieve the same lifestyle goals as the other participants also provided a source of collegial support.

### Program duration and follow-up

Based upon published outcomes of the American and Finnish Diabetes prevention studies [7,65], and other trials conducted in our research clinic [66] we considered that a 4 month period was sufficient to demonstrate the effect of the intervention on study outcome measures. In order to maximise the potential benefit of the intervention, but without over-burdening participants, education meetings were scheduled weekly for 1 hour with 45–60 min exercise following. Frequency of contact was adapted from the 'Lifestyle balance' program by the Diabetes Prevention Program Research Group (DPPRG)[40], the aims of which were weight loss and increased PA. Weekly contact in initial stages is accepted as standard for weight loss interventions [67]. These and other reports suggest that individuals are more likely to maintain lifestyle changes if they have regular follow-up [28]. [67-70]

### Program compliance

Assessments of compliance revealed that the program was well tolerated. The gradual decline in attendance appearing approximately half-way through may suggest 'program fatigue', but no further data is available to support this. Interventions of longer duration tend to have greater attrition [48] and more than 50% dropout might be expected in community-based interventions [19]. Although commitment to weekly sessions may have been at times difficult to manage for participants, it appears that these compliance rates are acceptable. To compare with other lifestyle interventions [15,48-50], we might expect average attendance of 75% to information, and 50% to exercise sessions, so attendance to our information sessions was consistent with other studies, and exercise adherence slightly better. Attendance to information tended to predict attendance to group exercise, which may indicate that the format of the program was useful in encouraging participation in exercise, as participants may have been more likely to join in group exercise or use the gym (directly after the information session). Some have suggested the use of home-based exercise equipment as a means to improving adherence in overweight adults, especially women [71] which appears to be consistent with the feedback reported by participants during focus groups. Some research indicates that although home-based training is not as effective at improving factors such as glycemic control, it is effective for maintaining improvements in muscle strength and lean body mass following gymnasium-based training [72].

Compliance with self-reports were poorly completed, with only one-third of participants returning a reasonable record of weekly PA and food serves consumed. Adherence to self-monitoring during lifestyle intervention is a complex issue and is notoriously sub-optimal [73], which is consistent with other weight-management studies [12,74].

### Program uptake/acceptability

Our understanding of participants' perceived lifestyle changes and reasons for being involved is limited to a small sample of responses. Despite this, some common themes were identified that appear to support the major aims of the program but this may be biased due to the most motivated individuals attending focus groups, and also because responses were collected during the intervention. For perceived lifestyle changes, most frequent responses were around specific dietary changes, and engagement in formal exercise (including resistance exercise and trying new activities) and incidental PA. Other interesting comments included feelings of self-discipline, self control, motivation and the formation of better habits. Although it is difficult to extrapolate from this small sample of responses, it appeared that the main motivations given for participating were not simply to 'lose weight', but to modify size, health risk factors and lifestyle habits. This may be suggesting that individuals taking on lifestyle changes were seeing a bigger picture than actually making dietary and activity adjustments.

### Mechanisms

Results from the focus-group indicated that there was significant value in certain aspects of the dietary education provided, including food-label reading, cooking, and nutritional composition of packaged foods. There was also interest in specific information on serving sizes and daily allowances, which may have benefitted the more motivated individuals to better understand their required energy intake. Participants also suggested more instruction for home-based exercises. Participants considered peer-leadership to be important and interactive sessions more valuable than a didactic format, and the peer-group setting was important for collegial support. Learning and applying self-management skills was also found to be useful. Clinical monitoring, progress checks and continued support were also identified as vital for continued motivation. The acknowledgement of psychological counselling is not a surprising finding owing to the complex nature of obesity and the underlying issues that present barriers to healthy lifestyle.

Participants commented that the session content was appropriate to the level of understanding of the participants, and participants consequently were empowered to make behavioural changes. Increased feelings of wellbeing and achievement were noted as a result. It was identified that there is a need to focus on maintenance and support of these behaviours after the cessation of the intervention, including clinical assessment and social support networks.

### A simpler strategy for lifestyle modification?

Community or population level interventions need to be simple and inexpensive to be cost effective. The Ottawa charter for health promotion (WHO) states that healthy choices should be the easy choices [75] and there is further evidence emerging that this may empower individuals to continue making the healthy choices [76]. This is an important consideration in this context, where the ultimate goal is to avoid preventable lifestyle disease. This study was predicated on the assumption that the usual dietary restriction and high levels of PA to counter overweight are difficult for the general population to sustain [77]. Our approach was to implement a program based on dietary and PA guidelines that were already in place, and to assess its effect on clinical outcomes in comparison to a control group who were simply provided with the guideline documents.

This study also confirms that simply providing written guidelines to CON was not sufficient to effect significant change in dietary and physical activity behaviour. The implication of this is that the current practice of government and other organisations to develop and disseminate written guidelines will not of itself address the needs of overweight individuals. Rather these guidelines are best accompanied by additional support strategies including active behavioural intervention and practical examples that translate guidelines into everyday strategies that assist individuals from different backgrounds in achieving lifestyle recommendations

To our knowledge, this is the first demonstration of a targeted self-management program for individuals who are overweight/obese with MetS, in a randomised controlled trial. Longer-term research is warranted to understand whether a peer-led model would translate into improvements in body composition, cardiovascular risk factors and quality of life, and whether it would be readily accepted in the community. The current intervention has achieved success in the short-term, and analysis of long term clinical and compliance data will contribute to current understandings of the effectiveness of 'modest' lifestyle intervention on body composition and metabolic fitness. The importance of active or follow-up in maintaining improvements will be of particular interest in this assessment. Further research may then be warranted to investigate the sustainability of a similar program in a peer led, community-based setting.

## Competing interests

The authors declare that they have no competing interests.

## Authors' contributions

All authors read, assisted in editing and approved the final manuscript. TLP drafted the manuscript, coordinated the design and conduct of the clinical study, co-created the intervention program, and conducted the intervention. GMHM was project manager, was involved conducting the intervention, and contributed to drafting and revising the manuscript. KO assisted in drafting and revision of the manuscript. KW assisted in coordination and conduct of the clinical study, co-created the intervention program, and conducted the intervention. AMC contributed to the clinical study design and drafting of the manuscript. JDB contributed to the clinical study design and revision of the manuscript. PRCH contributed to the clinical study design and revision of the manuscript
